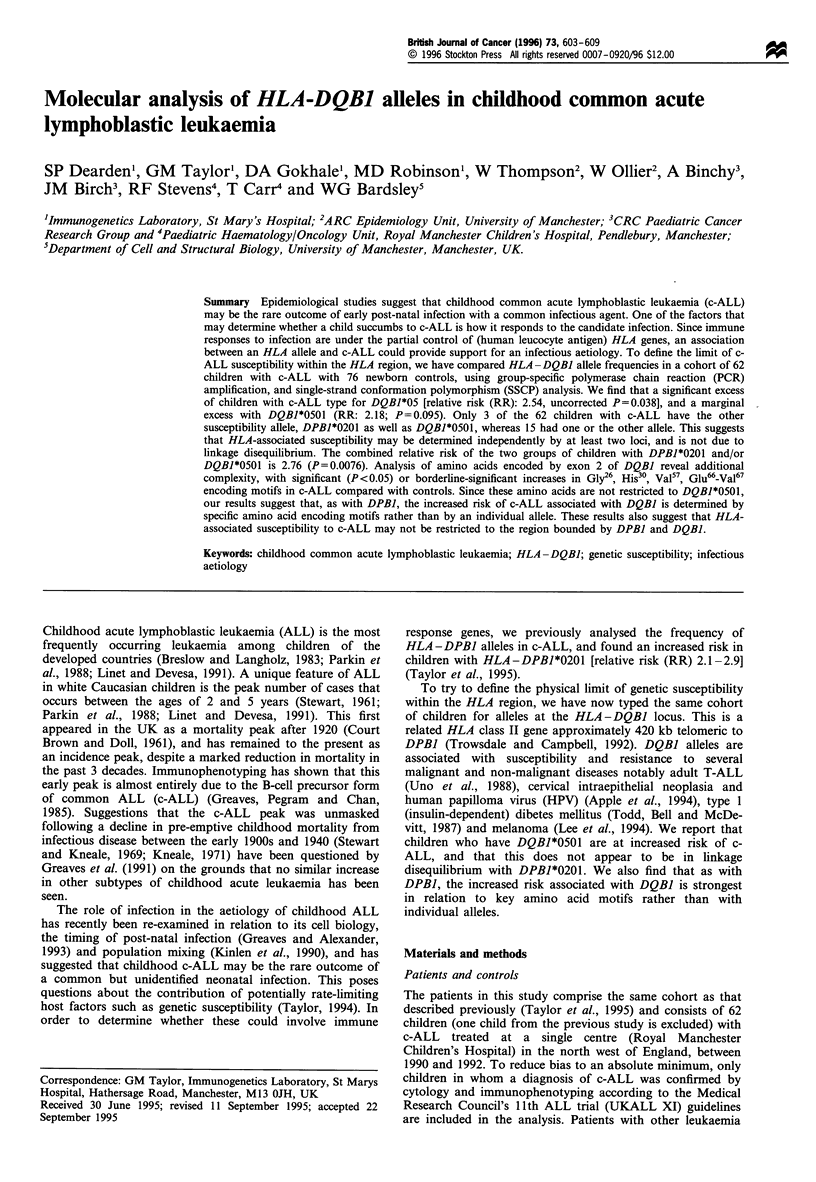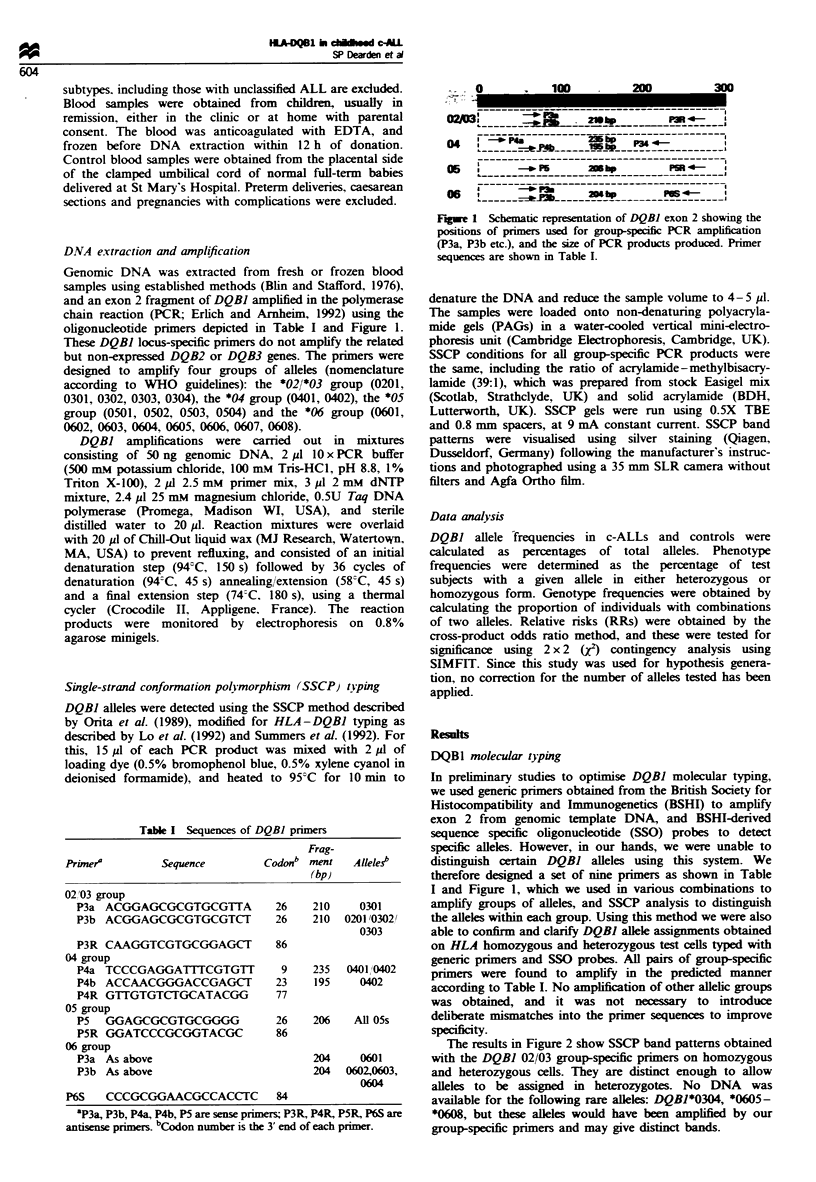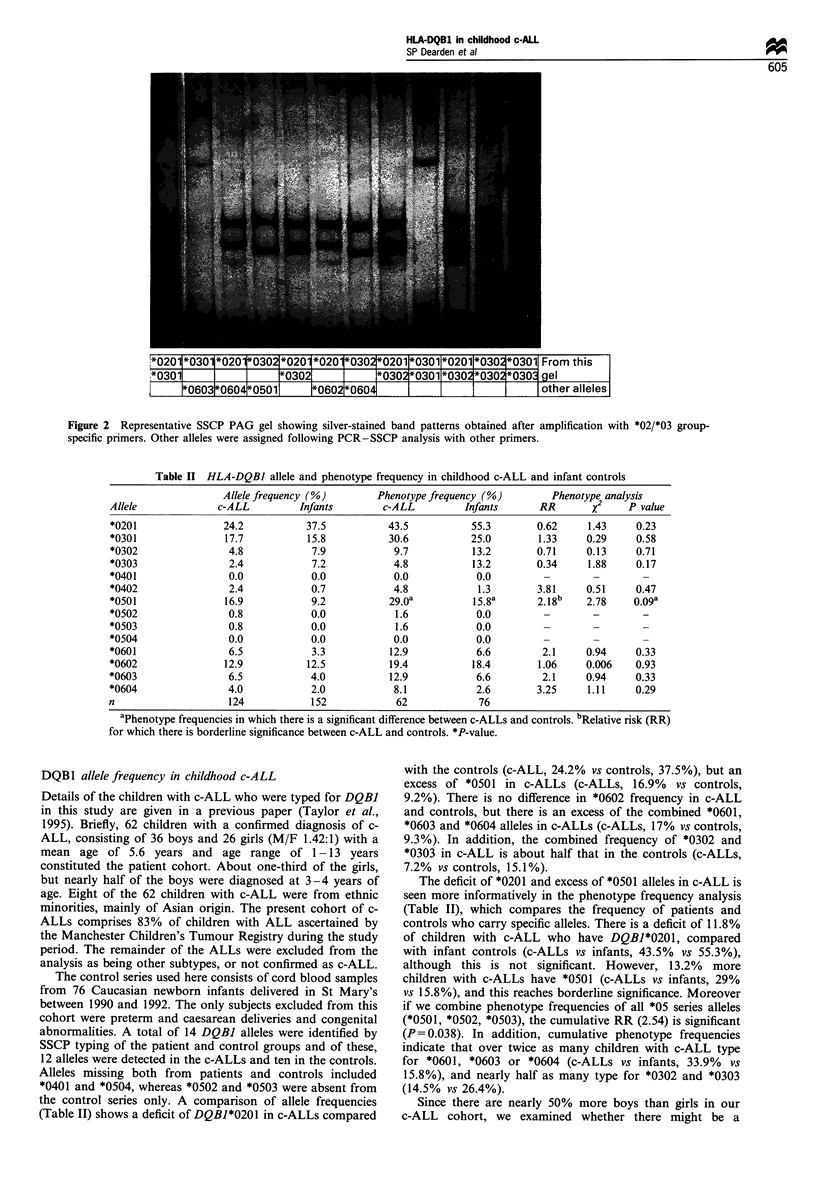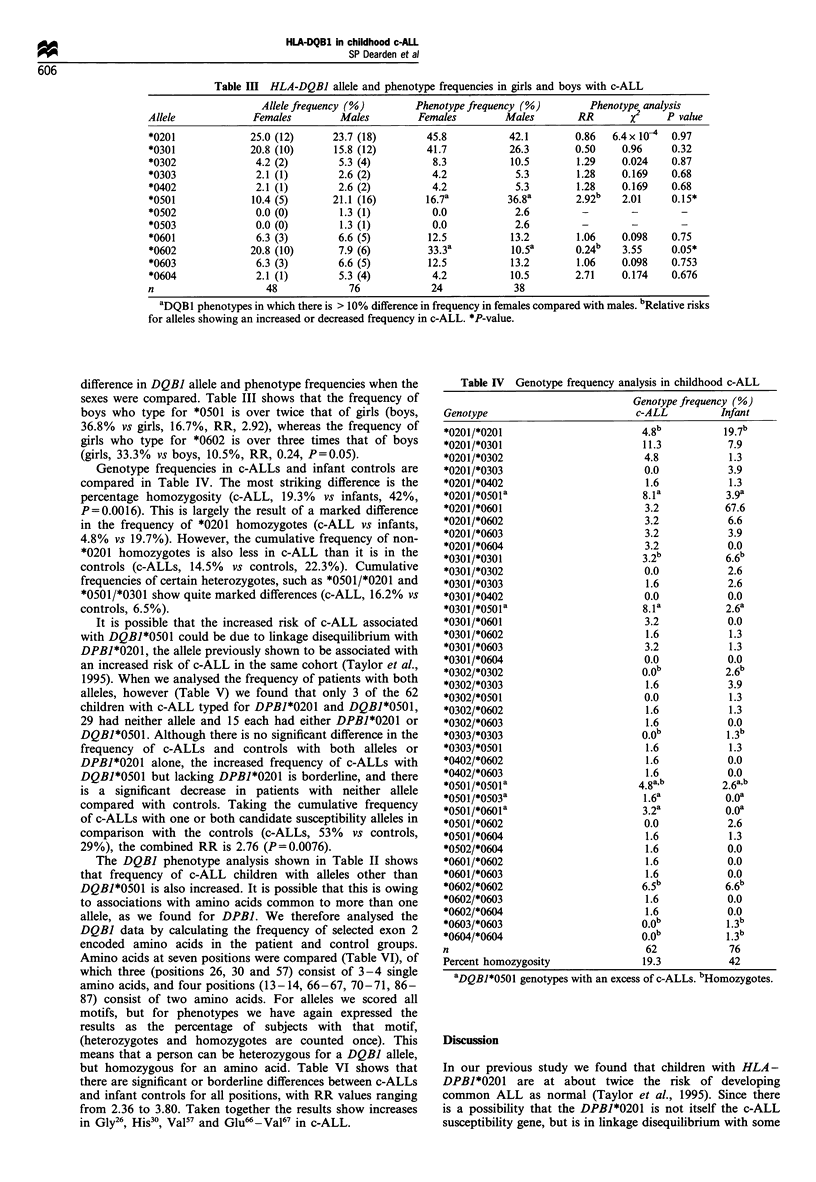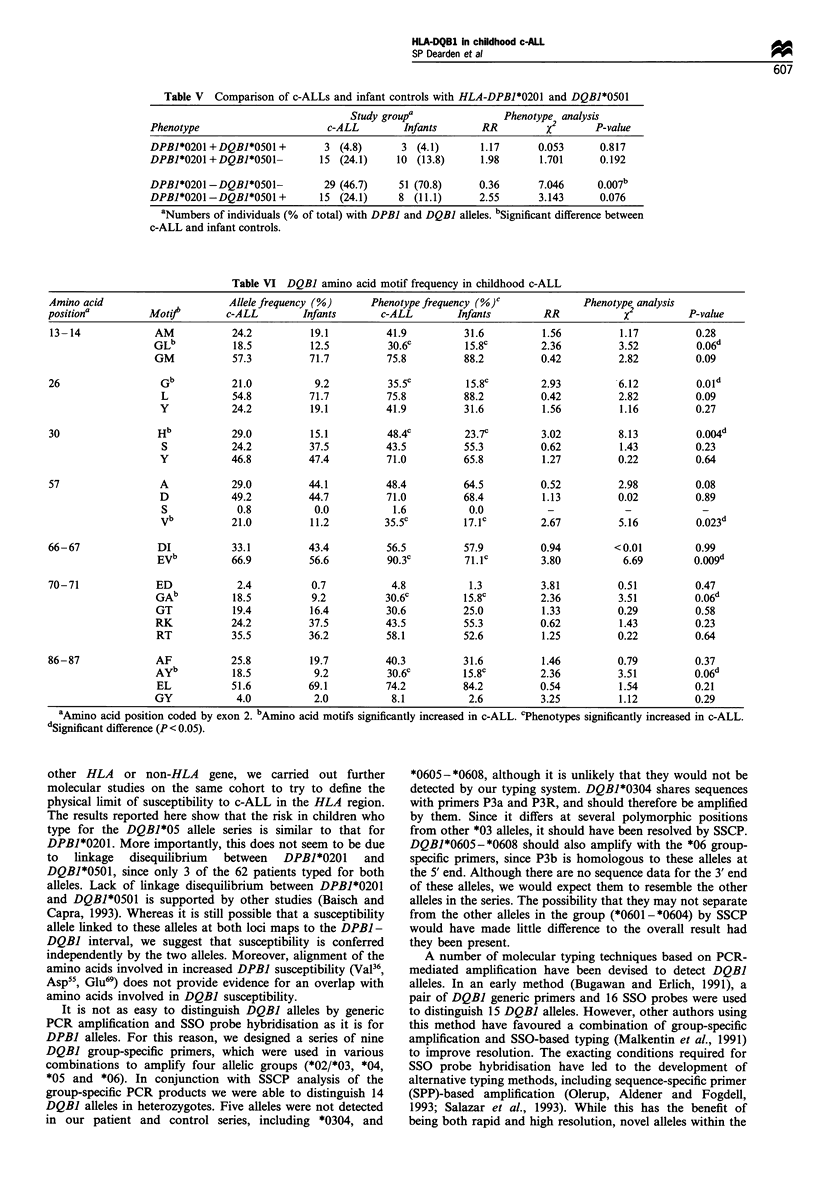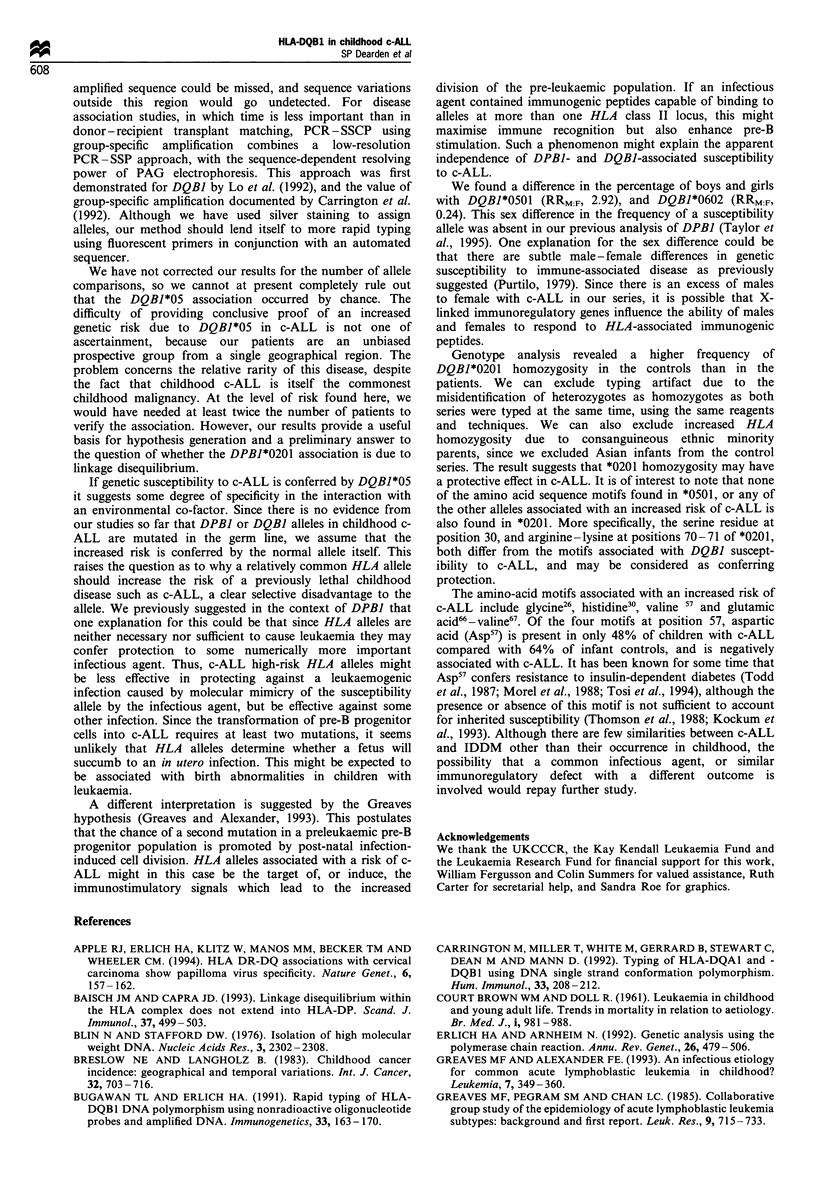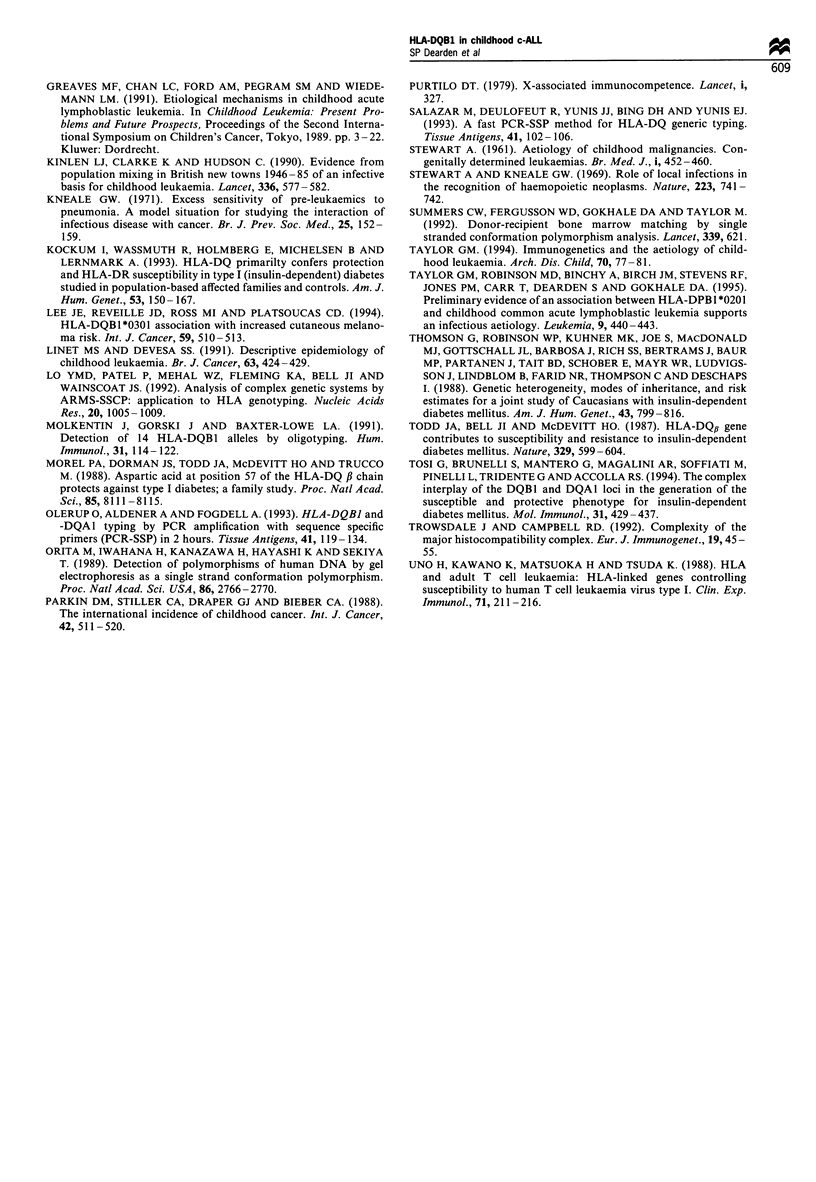# Molecular analysis of HLA-DQB1 alleles in childhood common acute lymphoblastic leukaemia.

**DOI:** 10.1038/bjc.1996.104

**Published:** 1996-03

**Authors:** S. P. Dearden, G. M. Taylor, D. A. Gokhale, M. D. Robinson, W. Thompson, W. Ollier, A. Binchy, J. M. Birch, R. F. Stevens, T. Carr, W. G. Bardsley

**Affiliations:** Immunogenetics Laboratory, St Mary's Hospital, Manchester, UK.

## Abstract

**Images:**


British Journal of Cancer (1996) 73, 603-609
© 1996 Stockton Press All rights reserved 0007-0920/96 $12.00

### Molecular analysis of *HLA-DQB1* alleles in childhood common acute lymphoblastic leukaemia

SP Dearden[1], GM Taylor[1], DA Gokhale[1], MD Robinson[1], W Thompson[2], W Ollier[2], A Binchy[3], JM Birch[3], RF Stevens[4], T Carr[4] and WG Bardsley[5]

[1]*Immunogenetics Laboratory, St Mary's Hospital;* [2]*ARC Epidemiology Unit, University of Manchester;* [3]*CRC Paediatric Cancer Research Group and* [4]*Paediatric Haematology/Oncology Unit, Royal Manchester Children's Hospital, Pendlebury, Manchester;* [5]*Department of Cell and Structural Biology, University of Manchester, Manchester, UK.*

**Summary** Epidemiological studies suggest that childhood common acute lymphoblastic leukaemia (c-ALL) may be the rare outcome of early post-natal infection with a common infectious agent. One of the factors that may determine whether a child succumbs to c-ALL is how it responds to the candidate infection. Since immune responses to infection are under the partial control of (human leucocyte antigen) *HLA* genes, an association between an *HLA* allele and c-ALL could provide support for an infectious aetiology. To define the limit of c-ALL susceptibility within the *HLA* region, we have compared *HLA-DQB1* allele frequencies in a cohort of 62 children with c-ALL with 76 newborn controls, using group-specific polymerase chain reaction (PCR) amplification, and single-strand conformation polymorphism (SSCP) analysis. We find that a significant excess of children with c-ALL type for *DQB1**05 [relative risk (RR): 2.54, uncorrected $P=0.038$], and a marginal excess with *DQB1**0501 (RR: 2.18; $P=0.095$). Only 3 of the 62 children with c-ALL have the other susceptibility allele, *DPB1**0201 as well as *DQB1**0501, whereas 15 had one or the other allele. This suggests that *HLA*-associated susceptibility may be determined independently by at least two loci, and is not due to linkage disequilibrium. The combined relative risk of the two groups of children with *DPB1**0201 and/or *DQB1**0501 is 2.76 ($P=0.0076$). Analysis of amino acids encoded by exon 2 of *DQB1* reveal additional complexity, with significant ($P<0.05$) or borderline-significant increases in $Gly^{26}$, $His^{30}$, $Val^{57}$, $Glu^{66}$-$Val^{67}$ encoding motifs in c-ALL compared with controls. Since these amino acids are not restricted to *DQB1**0501, our results suggest that, as with *DPB1*, the increased risk of c-ALL associated with *DQB1* is determined by specific amino acid encoding motifs rather than by an individual allele. These results also suggest that *HLA*-associated susceptibility to c-ALL may not be restricted to the region bounded by *DPB1* and *DQB1*.

**Keywords:** childhood common acute lymphoblastic leukaemia; *HLA-DQB1*; genetic susceptibility; infectious aetiology

Childhood acute lymphoblastic leukaemia (ALL) is the most frequently occurring leukaemia among children of the developed countries (Breslow and Langholz, 1983; Parkin *et al.*, 1988; Linet and Devesa, 1991). A unique feature of ALL in white Caucasian children is the peak number of cases that occurs between the ages of 2 and 5 years (Stewart, 1961; Parkin *et al.*, 1988; Linet and Devesa, 1991). This first appeared in the UK as a mortality peak after 1920 (Court Brown and Doll, 1961), and has remained to the present as an incidence peak, despite a marked reduction in mortality in the past 3 decades. Immunophenotyping has shown that this early peak is almost entirely due to the B-cell precursor form of common ALL (c-ALL) (Greaves, Pegram and Chan, 1985). Suggestions that the c-ALL peak was unmasked following a decline in pre-emptive childhood mortality from infectious disease between the early 1900s and 1940 (Stewart and Kneale, 1969; Kneale, 1971) have been questioned by Greaves *et al.* (1991) on the grounds that no similar increase in other subtypes of childhood acute leukaemia has been seen.

The role of infection in the aetiology of childhood ALL has recently been re-examined in relation to its cell biology, the timing of post-natal infection (Greaves and Alexander, 1993) and population mixing (Kinlen *et al.*, 1990), and has suggested that childhood c-ALL may be the rare outcome of a common but unidentified neonatal infection. This poses questions about the contribution of potentially rate-limiting host factors such as genetic susceptibility (Taylor, 1994). In order to determine whether these could involve immune response genes, we previously analysed the frequency of *HLA-DPB1* alleles in c-ALL, and found an increased risk in children with *HLA-DPB1**0201 [relative risk (RR) 2.1-2.9] (Taylor *et al.*, 1995).

To try to define the physical limit of genetic susceptibility within the *HLA* region, we have now typed the same cohort of children for alleles at the *HLA-DQB1* locus. This is a related *HLA* class II gene approximately 420 kb telomeric to *DPB1* (Trowsdale and Campbell, 1992). *DQB1* alleles are associated with susceptibility and resistance to several malignant and non-malignant diseases notably adult T-ALL (Uno *et al.*, 1988), cervical intraepithelial neoplasia and human papilloma virus (HPV) (Apple *et al.*, 1994), type 1 (insulin-dependent) dibetes mellitus (Todd, Bell and McDevitt, 1987) and melanoma (Lee *et al.*, 1994). We report that children who have *DQB1**0501 are at increased risk of c-ALL, and that this does not appear to be in linkage disequilibrium with *DPB1**0201. We also find that as with *DPB1*, the increased risk associated with *DQB1* is strongest in relation to key amino acid motifs rather than with individual alleles.

### Materials and methods

### *Patients and controls*

The patients in this study comprise the same cohort as that described previously (Taylor *et al.*, 1995) and consists of 62 children (one child from the previous study is excluded) with c-ALL treated at a single centre (Royal Manchester Children's Hospital) in the north west of England, between 1990 and 1992. To reduce bias to an absolute minimum, only children in whom a diagnosis of c-ALL was confirmed by cytology and immunophenotyping according to the Medical Research Council's 11th ALL trial (UKALL XI) guidelines are included in the analysis. Patients with other leukaemia

Correspondence: GM Taylor, Immunogenetics Laboratory, St Marys Hospital, Hathersage Road, Manchester, M13 0JH, UK
Received 30 June 1995; revised 11 September 1995; accepted 22 September 1995

subtypes, including those with unclassified ALL are excluded. Blood samples were obtained from children, usually in remission, either in the clinic or at home with parental consent. The blood was anticoagulated with EDTA, and frozen before DNA extraction within 12 h of donation. Control blood samples were obtained from the placental side of the clamped umbilical cord of normal full-term babies delivered at St Mary's Hospital. Preterm deliveries, caesarean sections and pregnancies with complications were excluded.

### *DNA extraction and amplification*

Genomic DNA was extracted from fresh or frozen blood samples using established methods (Blin and Stafford, 1976), and an exon 2 fragment of *DQB1* amplified in the polymerase chain reaction (PCR; Erlich and Arnheim, 1992) using the oligonucleotide primers depicted in Table I and Figure 1. These *DQB1* locus-specific primers do not amplify the related but non-expressed *DQB2* or *DQB3* genes. The primers were designed to amplify four groups of alleles (nomenclature according to WHO guidelines): the **02/*03* group (0201, 0301, 0302, 0303, 0304), the **04* group (0401, 0402), the **05* group (0501, 0502, 0503, 0504) and the **06* group (0601, 0602, 0603, 0604, 0605, 0606, 0607, 0608).

*DQB1* amplifications were carried out in mixtures consisting of 50 ng genomic DNA, 2 μl 10×PCR buffer (500 mM potassium chloride, 100 mM Tris-HCl, pH 8.8, 1% Triton X-100), 2 μl 2.5 mM primer mix, 3 μl 2 mM dNTP mixture, 2.4 μl 25 mM magnesium chloride, 0.5U *Taq* DNA polymerase (Promega, Madison WI, USA), and sterile distilled water to 20 μl. Reaction mixtures were overlaid with 20 μl of Chill-Out liquid wax (MJ Research, Watertown, MA, USA) to prevent refluxing, and consisted of an initial denaturation step (94°C, 150 s) followed by 36 cycles of denaturation (94°C, 45 s) annealing/extension (58°C, 45 s) and a final extension step (74°C, 180 s), using a thermal cycler (Crocodile II, Appligene, France). The reaction products were monitored by electrophoresis on 0.8% agarose minigels.

### *Single-strand conformation polymorphism (SSCP) typing*

*DQB1* alleles were detected using the SSCP method described by Orita *et al.* (1989), modified for *HLA–DQB1* typing as described by Lo *et al.* (1992) and Summers *et al.* (1992). For this, 15 μl of each PCR product was mixed with 2 μl of loading dye (0.5% bromophenol blue, 0.5% xylene cyanol in deionised formamide), and heated to 95°C for 10 min to

**Table I** Sequences of *DQB1* primers

| Primer[a] | Sequence | Codon[b] | Frag-ment (bp) | Alleles[b] |
|---|---|---|---|---|
| 02/03 group | | | | |
| P3a | ACGGAGCGCGTGCGTTA | 26 | 210 | 0301 |
| P3b | ACGGAGCGCGTGCGTCT | 26 | 210 | 0201/0302/0303 |
| P3R | CAAGGTCGTGCGGAGCT | 86 | | |
| 04 group | | | | |
| P4a | TCCCGAGGATTTCGTGTT | 9 | 235 | 0401/0402 |
| P4b | ACCAACGGGACCGAGCT | 23 | 195 | 0402 |
| P4R | GTTGTGTCTGCATACGG | 77 | | |
| 05 group | | | | |
| P5 | GGAGCGCGTGCGGGG | 26 | 206 | All 05s |
| P5R | GGATCCCGCGGTACGC | 86 | | |
| 06 group | | | | |
| P3a | As above | | 204 | 0601 |
| P3b | As above | | 204 | 0602,0603, 0604 |
| P6S | CCCGCGGAACGCCACCTC | 84 | | |

[a]P3a, P3b, P4a, P4b, P5 are sense primers; P3R, P4R, P5R, P6S are antisense primers. [b]Codon number is the 3′ end of each primer.

**Figure 1** Schematic representation of *DQB1* exon 2 showing the positions of primers used for group-specific PCR amplification (P3a, P3b etc.), and the size of PCR products produced. Primer sequences are shown in Table I.

denature the DNA and reduce the sample volume to 4–5 μl. The samples were loaded onto non-denaturing polyacrylamide gels (PAGs) in a water-cooled vertical mini-electrophoresis unit (Cambridge Electrophoresis, Cambridge, UK). SSCP conditions for all group-specific PCR products were the same, including the ratio of acrylamide–methylbisacrylamide (39:1), which was prepared from stock Easigel mix (Scotlab, Strathclyde, UK) and solid acrylamide (BDH, Lutterworth, UK). SSCP gels were run using 0.5X TBE and 0.8 mm spacers, at 9 mA constant current. SSCP band patterns were visualised using silver staining (Qiagen, Dusseldorf, Germany) following the manufacturer's instructions and photographed using a 35 mm SLR camera without filters and Agfa Ortho film.

### *Data analysis*

*DQB1* allele frequencies in c-ALLs and controls were calculated as percentages of total alleles. Phenotype frequencies were determined as the percentage of test subjects with a given allele in either heterozygous or homozygous form. Genotype frequencies were obtained by calculating the proportion of individuals with combinations of two alleles. Relative risks (RRs) were obtained by the cross-product odds ratio method, and these were tested for significance using 2×2 ($\chi^2$) contingency analysis using SIMFIT. Since this study was used for hypothesis generation, no correction for the number of alleles tested has been applied.

### Results

### DQB1 *molecular typing*

In preliminary studies to optimise *DQB1* molecular typing, we used generic primers obtained from the British Society for Histocompatibility and Immunogenetics (BSHI) to amplify exon 2 from genomic template DNA, and BSHI-derived sequence specific oligonucleotide (SSO) probes to detect specific alleles. However, in our hands, we were unable to distinguish certain *DQB1* alleles using this system. We therefore designed a set of nine primers as shown in Table I and Figure 1, which we used in various combinations to amplify groups of alleles, and SSCP analysis to distinguish the alleles within each group. Using this method we were also able to confirm and clarify *DQB1* allele assignments obtained on *HLA* homozygous and heterozygous test cells typed with generic primers and SSO probes. All pairs of group-specific primers were found to amplify in the predicted manner according to Table I. No amplification of other allelic groups was obtained, and it was not necessary to introduce deliberate mismatches into the primer sequences to improve specificity.

The results in Figure 2 show SSCP band patterns obtained with the *DQB1* 02/03 group-specific primers on homozygous and heterozygous cells. They are distinct enough to allow alleles to be assigned in heterozygotes. No DNA was available for the following rare alleles: *DQB1**0304, *0605–*0608, but these alleles would have been amplified by our group-specific primers and may give distinct bands.

**Figure 2** Representative SSCP PAG gel showing silver-stained band patterns obtained after amplification with *02/*03 group-specific primers. Other alleles were assigned following PCR–SSCP analysis with other primers.

**Table II** *HLA-DQB1* allele and phenotype frequency in childhood c-ALL and infant controls

| *Allele* | *Allele frequency (%)* c-ALL | *Infants* | *Phenotype frequency (%)* c-ALL | *Infants* | *Phenotype analysis* RR | $\chi^2$ | *P value* |
|---|---|---|---|---|---|---|---|
| *0201 | 24.2 | 37.5 | 43.5 | 55.3 | 0.62 | 1.43 | 0.23 |
| *0301 | 17.7 | 15.8 | 30.6 | 25.0 | 1.33 | 0.29 | 0.58 |
| *0302 | 4.8 | 7.9 | 9.7 | 13.2 | 0.71 | 0.13 | 0.71 |
| *0303 | 2.4 | 7.2 | 4.8 | 13.2 | 0.34 | 1.88 | 0.17 |
| *0401 | 0.0 | 0.0 | 0.0 | 0.0 | – | – | – |
| *0402 | 2.4 | 0.7 | 4.8 | 1.3 | 3.81 | 0.51 | 0.47 |
| *0501 | 16.9 | 9.2 | 29.0[a] | 15.8[a] | 2.18[b] | 2.78 | 0.09[a] |
| *0502 | 0.8 | 0.0 | 1.6 | 0.0 | – | – | – |
| *0503 | 0.8 | 0.0 | 1.6 | 0.0 | – | – | – |
| *0504 | 0.0 | 0.0 | 0.0 | 0.0 | – | – | – |
| *0601 | 6.5 | 3.3 | 12.9 | 6.6 | 2.1 | 0.94 | 0.33 |
| *0602 | 12.9 | 12.5 | 19.4 | 18.4 | 1.06 | 0.006 | 0.93 |
| *0603 | 6.5 | 4.0 | 12.9 | 6.6 | 2.1 | 0.94 | 0.33 |
| *0604 | 4.0 | 2.0 | 8.1 | 2.6 | 3.25 | 1.11 | 0.29 |
| *n* | 124 | 152 | 62 | 76 | | | |

[a]Phenotype frequencies in which there is a significant difference between c-ALLs and controls. [b]Relative risk (RR) for which there is borderline significance between c-ALL and controls. **P*-value.

### DQB1 *allele frequency in childhood c-ALL*

Details of the children with c-ALL who were typed for *DQB1* in this study are given in a previous paper (Taylor *et al.*, 1995). Briefly, 62 children with a confirmed diagnosis of c-ALL, consisting of 36 boys and 26 girls (M/F 1.42:1) with a mean age of 5.6 years and age range of 1–13 years constituted the patient cohort. About one-third of the girls, but nearly half of the boys were diagnosed at 3–4 years of age. Eight of the 62 children with c-ALL were from ethnic minorities, mainly of Asian origin. The present cohort of c-ALLs comprises 83% of children with ALL ascertained by the Manchester Children's Tumour Registry during the study period. The remainder of the ALLs were excluded from the analysis as being other subtypes, or not confirmed as c-ALL.

The control series used here consists of cord blood samples from 76 Caucasian newborn infants delivered in St Mary's between 1990 and 1992. The only subjects excluded from this cohort were preterm and caesarean deliveries and congenital abnormalities. A total of 14 *DQB1* alleles were identified by SSCP typing of the patient and control groups and of these, 12 alleles were detected in the c-ALLs and ten in the controls. Alleles missing both from patients and controls included *0401 and *0504, whereas *0502 and *0503 were absent from the control series only. A comparison of allele frequencies (Table II) shows a deficit of *DQB1**0201 in c-ALLs compared with the controls (c-ALL, 24.2% *vs* controls, 37.5%), but an excess of *0501 in c-ALLs (c-ALLs, 16.9% *vs* controls, 9.2%). There is no difference in *0602 frequency in c-ALL and controls, but there is an excess of the combined *0601, *0603 and *0604 alleles in c-ALLs (c-ALLs, 17% *vs* controls, 9.3%). In addition, the combined frequency of *0302 and *0303 in c-ALL is about half that in the controls (c-ALLs, 7.2% *vs* controls, 15.1%).

The deficit of *0201 and excess of *0501 alleles in c-ALL is seen more informatively in the phenotype frequency analysis (Table II), which compares the frequency of patients and controls who carry specific alleles. There is a deficit of 11.8% of children with c-ALL who have *DQB1**0201, compared with infant controls (c-ALLs *vs* infants, 43.5% *vs* 55.3%), although this is not significant. However, 13.2% more children with c-ALLs have *0501 (c-ALLs *vs* infants, 29% *vs* 15.8%), and this reaches borderline significance. Moreover if we combine phenotype frequencies of all *05 series alleles (*0501, *0502, *0503), the cumulative RR (2.54) is significant ($P = 0.038$). In addition, cumulative phenotype frequencies indicate that over twice as many children with c-ALL type for *0601, *0603 or *0604 (c-ALLs *vs* infants, 33.9% *vs* 15.8%), and nearly half as many type for *0302 and *0303 (14.5% *vs* 26.4%).

Since there are nearly 50% more boys than girls in our c-ALL cohort, we examined whether there might be a

**Table III** *HLA-DQB1* allele and phenotype frequencies in girls and boys with c-ALL

| *Allele* | *Allele frequency (%)* *Females* | *Males* | *Phenotype frequency (%)* *Females* | *Males* | *Phenotype analysis* *RR* | $\chi^2$ | *P value* |
|---|---|---|---|---|---|---|---|
| *0201 | 25.0 (12) | 23.7 (18) | 45.8 | 42.1 | 0.86 | $6.4\times10^{-4}$ | 0.97 |
| *0301 | 20.8 (10) | 15.8 (12) | 41.7 | 26.3 | 0.50 | 0.96 | 0.32 |
| *0302 | 4.2 (2) | 5.3 (4) | 8.3 | 10.5 | 1.29 | 0.024 | 0.87 |
| *0303 | 2.1 (1) | 2.6 (2) | 4.2 | 5.3 | 1.28 | 0.169 | 0.68 |
| *0402 | 2.1 (1) | 2.6 (2) | 4.2 | 5.3 | 1.28 | 0.169 | 0.68 |
| *0501 | 10.4 (5) | 21.1 (16) | 16.7[a] | 36.8[a] | 2.92[b] | 2.01 | 0.15* |
| *0502 | 0.0 (0) | 1.3 (1) | 0.0 | 2.6 | – | – | – |
| *0503 | 0.0 (0) | 1.3 (1) | 0.0 | 2.6 | – | – | – |
| *0601 | 6.3 (3) | 6.6 (5) | 12.5 | 13.2 | 1.06 | 0.098 | 0.75 |
| *0602 | 20.8 (10) | 7.9 (6) | 33.3[a] | 10.5[a] | 0.24[b] | 3.55 | 0.05* |
| *0603 | 6.3 (3) | 6.6 (5) | 12.5 | 13.2 | 1.06 | 0.098 | 0.753 |
| *0604 | 2.1 (1) | 5.3 (4) | 4.2 | 10.5 | 2.71 | 0.174 | 0.676 |
| *n* | 48 | 76 | 24 | 38 | | | |

[a]DQB1 phenotypes in which there is > 10% difference in frequency in females compared with males. [b]Relative risks for alleles showing an increased or decreased frequency in c-ALL. **P*-value.

difference in *DQB1* allele and phenotype frequencies when the sexes were compared. Table III shows that the frequency of boys who type for *0501 is over twice that of girls (boys, 36.8% *vs* girls, 16.7%, RR, 2.92), whereas the frequency of girls who type for *0602 is over three times that of boys (girls, 33.3% *vs* boys, 10.5%, RR, 0.24, $P=0.05$).

Genotype frequencies in c-ALLs and infant controls are compared in Table IV. The most striking difference is the percentage homozygosity (c-ALL, 19.3% *vs* infants, 42%, $P=0.0016$). This is largely the result of a marked difference in the frequency of *0201 homozygotes (c-ALL *vs* infants, 4.8% *vs* 19.7%). However, the cumulative frequency of non-*0201 homozygotes is also less in c-ALL than it is in the controls (c-ALLs, 14.5% *vs* controls, 22.3%). Cumulative frequencies of certain heterozygotes, such as *0501/*0201 and *0501/*0301 show quite marked differences (c-ALL, 16.2% *vs* controls, 6.5%).

It is possible that the increased risk of c-ALL associated with *DQB1**0501 could be due to linkage disequilibrium with *DPB1**0201, the allele previously shown to be associated with an increased risk of c-ALL in the same cohort (Taylor *et al.*, 1995). When we analysed the frequency of patients with both alleles, however (Table V) we found that only 3 of the 62 children with c-ALL typed for *DPB1**0201 and *DQB1**0501, 29 had neither allele and 15 each had either *DPB1**0201 or *DQB1**0501. Although there is no significant difference in the frequency of c-ALLs and controls with both alleles or *DPB1**0201 alone, the increased frequency of c-ALLs with *DQB1**0501 but lacking *DPB1**0201 is borderline, and there is a significant decrease in patients with neither allele compared with controls. Taking the cumulative frequency of c-ALLs with one or both candidate susceptibility alleles in comparison with the controls (c-ALLs, 53% *vs* controls, 29%), the combined RR is 2.76 ($P=0.0076$).

The *DQB1* phenotype analysis shown in Table II shows that frequency of c-ALL children with alleles other than *DQB1**0501 is also increased. It is possible that this is owing to associations with amino acids common to more than one allele, as we found for *DPB1*. We therefore analysed the *DQB1* data by calculating the frequency of selected exon 2 encoded amino acids in the patient and control groups. Amino acids at seven positions were compared (Table VI), of which three (positions 26, 30 and 57) consist of 3–4 single amino acids, and four positions (13–14, 66–67, 70–71, 86–87) consist of two amino acids. For alleles we scored all motifs, but for phenotypes we have again expressed the results as the percentage of subjects with that motif, (heterozygotes and homozygotes are counted once). This means that a person can be heterozygous for a *DQB1* allele, but homozygous for an amino acid. Table VI shows that there are significant or borderline differences between c-ALLs and infant controls for all positions, with RR values ranging from 2.36 to 3.80. Taken together the results show increases in $Gly^{26}$, $His^{30}$, $Val^{57}$ and $Glu^{66}$–$Val^{67}$ in c-ALL.

**Table IV** Genotype frequency analysis in childhood c-ALL

| *Genotype* | *Genotype frequency (%)* *c-ALL* | *Infant* |
|---|---|---|
| *0201/*0201 | 4.8[b] | 19.7[b] |
| *0201/*0301 | 11.3 | 7.9 |
| *0201/*0302 | 4.8 | 1.3 |
| *0201/*0303 | 0.0 | 3.9 |
| *0201/*0402 | 1.6 | 1.3 |
| *0201/*0501[a] | 8.1[a] | 3.9[a] |
| *0201/*0601 | 3.2 | 67.6 |
| *0201/*0602 | 3.2 | 6.6 |
| *0201/*0603 | 3.2 | 3.9 |
| *0201/*0604 | 3.2 | 0.0 |
| *0301/*0301 | 3.2[b] | 6.6[b] |
| *0301/*0302 | 0.0 | 2.6 |
| *0301/*0303 | 1.6 | 2.6 |
| *0301/*0402 | 0.0 | 0.0 |
| *0301/*0501[a] | 8.1[a] | 2.6[a] |
| *0301/*0601 | 3.2 | 0.0 |
| *0301/*0602 | 1.6 | 1.3 |
| *0301/*0603 | 3.2 | 1.3 |
| *0301/*0604 | 0.0 | 0.0 |
| *0302/*0302 | 0.0[b] | 2.6[b] |
| *0302/*0303 | 1.6 | 3.9 |
| *0302/*0501 | 0.0 | 1.3 |
| *0302/*0602 | 1.6 | 1.3 |
| *0302/*0603 | 1.6 | 0.0 |
| *0303/*0303 | 0.0[b] | 1.3[b] |
| *0303/*0501 | 1.6 | 1.3 |
| *0402/*0602 | 1.6 | 0.0 |
| *0402/*0603 | 1.6 | 0.0 |
| *0501/*0501[a] | 4.8[a,b] | 2.6[a,b] |
| *0501/*0503[a] | 1.6[a] | 0.0[a] |
| *0501/*0601[a] | 3.2[a] | 0.0[a] |
| *0501/*0602 | 0.0 | 2.6 |
| *0501/*0604 | 1.6 | 1.3 |
| *0502/*0604 | 1.6 | 0.0 |
| *0601/*0602 | 1.6 | 0.0 |
| *0601/*0603 | 1.6 | 0.0 |
| *0602/*0602 | 6.5[b] | 6.6[b] |
| *0602/*0603 | 1.6 | 0.0 |
| *0602/*0604 | 1.6 | 0.0 |
| *0603/*0603 | 0.0[b] | 1.3[b] |
| *0604/*0604 | 0.0[b] | 1.3[b] |
| *n* | 62 | 76 |
| Percent homozygosity | 19.3 | 42 |

[a]*DQB1**0501 genotypes with an excess of c-ALLs. [b]Homozygotes.

### Discussion

In our previous study we found that children with *HLA-DPB1**0201 are at about twice the risk of developing common ALL as normal (Taylor *et al.*, 1995). Since there is a possibility that the *DPB1**0201 is not itself the c-ALL susceptibility gene, but is in linkage disequilibrium with some

Table V Comparison of c-ALLs and infant controls with *HLA-DPB1**0201 and *DQB1**0501

| | Study group[a] | | Phenotype analysis | | |
|---|---|---|---|---|---|
| *Phenotype* | *c-ALL* | *Infants* | *RR* | $\chi^2$ | *P-value* |
| *DPB1**0201 + *DQB1**0501 + | 3 (4.8) | 3 (4.1) | 1.17 | 0.053 | 0.817 |
| *DPB1**0201 + *DQB1**0501– | 15 (24.1) | 10 (13.8) | 1.98 | 1.701 | 0.192 |
| *DPB1**0201 – *DQB1**0501– | 29 (46.7) | 51 (70.8) | 0.36 | 7.046 | 0.007[b] |
| *DPB1**0201 – *DQB1**0501 + | 15 (24.1) | 8 (11.1) | 2.55 | 3.143 | 0.076 |

[a]Numbers of individuals (% of total) with *DPB1* and *DQB1* alleles. [b]Significant difference between c-ALL and infant controls.

Table VI *DQB1* amino acid motif frequency in childhood c-ALL

| *Amino acid position*[a] | *Motif*[b] | Allele frequency (%) *c-ALL* | *Infants* | Phenotype frequency (%)[c] *c-ALL* | *Infants* | *RR* | Phenotype analysis $\chi^2$ | *P-value* |
|---|---|---|---|---|---|---|---|---|
| 13–14 | AM | 24.2 | 19.1 | 41.9 | 31.6 | 1.56 | 1.17 | 0.28 |
| | GL[b] | 18.5 | 12.5 | 30.6[c] | 15.8[c] | 2.36 | 3.52 | 0.06[d] |
| | GM | 57.3 | 71.7 | 75.8 | 88.2 | 0.42 | 2.82 | 0.09 |
| 26 | G[b] | 21.0 | 9.2 | 35.5[c] | 15.8[c] | 2.93 | 6.12 | 0.01[d] |
| | L | 54.8 | 71.7 | 75.8 | 88.2 | 0.42 | 2.82 | 0.09 |
| | Y | 24.2 | 19.1 | 41.9 | 31.6 | 1.56 | 1.16 | 0.27 |
| 30 | H[b] | 29.0 | 15.1 | 48.4[c] | 23.7[c] | 3.02 | 8.13 | 0.004[d] |
| | S | 24.2 | 37.5 | 43.5 | 55.3 | 0.62 | 1.43 | 0.23 |
| | Y | 46.8 | 47.4 | 71.0 | 65.8 | 1.27 | 0.22 | 0.64 |
| 57 | A | 29.0 | 44.1 | 48.4 | 64.5 | 0.52 | 2.98 | 0.08 |
| | D | 49.2 | 44.7 | 71.0 | 68.4 | 1.13 | 0.02 | 0.89 |
| | S | 0.8 | 0.0 | 1.6 | 0.0 | – | – | – |
| | V[b] | 21.0 | 11.2 | 35.5[c] | 17.1[c] | 2.67 | 5.16 | 0.023[d] |
| 66–67 | DI | 33.1 | 43.4 | 56.5 | 57.9 | 0.94 | < 0.01 | 0.99 |
| | EV[b] | 66.9 | 56.6 | 90.3[c] | 71.1[c] | 3.80 | 6.69 | 0.009[d] |
| 70–71 | ED | 2.4 | 0.7 | 4.8 | 1.3 | 3.81 | 0.51 | 0.47 |
| | GA[b] | 18.5 | 9.2 | 30.6[c] | 15.8[c] | 2.36 | 3.51 | 0.06[d] |
| | GT | 19.4 | 16.4 | 30.6 | 25.0 | 1.33 | 0.29 | 0.58 |
| | RK | 24.2 | 37.5 | 43.5 | 55.3 | 0.62 | 1.43 | 0.23 |
| | RT | 35.5 | 36.2 | 58.1 | 52.6 | 1.25 | 0.22 | 0.64 |
| 86–87 | AF | 25.8 | 19.7 | 40.3 | 31.6 | 1.46 | 0.79 | 0.37 |
| | AY[b] | 18.5 | 9.2 | 30.6[c] | 15.8[c] | 2.36 | 3.51 | 0.06[d] |
| | EL | 51.6 | 69.1 | 74.2 | 84.2 | 0.54 | 1.54 | 0.21 |
| | GY | 4.0 | 2.0 | 8.1 | 2.6 | 3.25 | 1.12 | 0.29 |

[a]Amino acid position coded by exon 2. [b]Amino acid motifs significantly increased in c-ALL. [c]Phenotypes significantly increased in c-ALL. [d]Significant difference ($P < 0.05$).

other *HLA* or non-*HLA* gene, we carried out further molecular studies on the same cohort to try to define the physical limit of susceptibility to c-ALL in the *HLA* region. The results reported here show that the risk in children who type for the *DQB1**05 allele series is similar to that for *DPB1**0201. More importantly, this does not seem to be due to linkage disequilibrium between *DPB1**0201 and *DQB1**0501, since only 3 of the 62 patients typed for both alleles. Lack of linkage disequilibrium between *DPB1**0201 and *DQB1**0501 is supported by other studies (Baisch and Capra, 1993). Whereas it is still possible that a susceptibility allele linked to these alleles at both loci maps to the *DPB1–DQB1* interval, we suggest that susceptibility is conferred independently by the two alleles. Moreover, alignment of the amino acids involved in increased *DPB1* susceptibility ($Val^{36}$, $Asp^{55}$, $Glu^{69}$) does not provide evidence for an overlap with amino acids involved in *DQB1* susceptibility.

It is not as easy to distinguish *DQB1* alleles by generic PCR amplification and SSO probe hybridisation as it is for *DPB1* alleles. For this reason, we designed a series of nine *DQB1* group-specific primers, which were used in various combinations to amplify four allelic groups (*02/*03, *04, *05 and *06). In conjunction with SSCP analysis of the group-specific PCR products we were able to distinguish 14 *DQB1* alleles in heterozygotes. Five alleles were not detected in our patient and control series, including *0304, and *0605 – *0608, although it is unlikely that they would not be detected by our typing system. *DQB1**0304 shares sequences with primers P3a and P3R, and should therefore be amplified by them. Since it differs at several polymorphic positions from other *03 alleles, it should have been resolved by SSCP. *DQB1**0605 – *0608 should also amplify with the *06 group-specific primers, since P3b is homologous to these alleles at the 5′ end. Although there are no sequence data for the 3′ end of these alleles, we would expect them to resemble the other alleles in the series. The possibility that they may not separate from the other alleles in the group (*0601 – *0604) by SSCP would have made little difference to the overall result had they been present.

A number of molecular typing techniques based on PCR-mediated amplification have been devised to detect *DQB1* alleles. In an early method (Bugawan and Erlich, 1991), a pair of *DQB1* generic primers and 16 SSO probes were used to distinguish 15 *DQB1* alleles. However, other authors using this method have favoured a combination of group-specific amplification and SSO-based typing (Malkentin *et al.*, 1991) to improve resolution. The exacting conditions required for SSO probe hybridisation have led to the development of alternative typing methods, including sequence-specific primer (SPP)-based amplification (Olerup, Aldener and Fogdell, 1993; Salazar *et al.*, 1993). While this has the benefit of being both rapid and high resolution, novel alleles within the amplified sequence could be missed, and sequence variations outside this region would go undetected. For disease association studies, in which time is less important than in donor–recipient transplant matching, PCR–SSCP using group-specific amplification combines a low-resolution PCR–SSP approach, with the sequence-dependent resolving power of PAG electrophoresis. This approach was first demonstrated for *DQB1* by Lo *et al.* (1992), and the value of group-specific amplification documented by Carrington *et al.* (1992). Although we have used silver staining to assign alleles, our method should lend itself to more rapid typing using fluorescent primers in conjunction with an automated sequencer.

We have not corrected our results for the number of allele comparisons, so we cannot at present completely rule out that the *DQB1**05 association occurred by chance. The difficulty of providing conclusive proof of an increased genetic risk due to *DQB1**05 in c-ALL is not one of ascertainment, because our patients are an unbiased prospective group from a single geographical region. The problem concerns the relative rarity of this disease, despite the fact that childhood c-ALL is itself the commonest childhood malignancy. At the level of risk found here, we would have needed at least twice the number of patients to verify the association. However, our results provide a useful basis for hypothesis generation and a preliminary answer to the question of whether the *DPB1**0201 association is due to linkage disequilibrium.

If genetic susceptibility to c-ALL is conferred by *DQB1**05 it suggests some degree of specificity in the interaction with an environmental co-factor. Since there is no evidence from our studies so far that *DPB1* or *DQB1* alleles in childhood c-ALL are mutated in the germ line, we assume that the increased risk is conferred by the normal allele itself. This raises the question as to why a relatively common *HLA* allele should increase the risk of a previously lethal childhood disease such as c-ALL, a clear selective disadvantage to the allele. We previously suggested in the context of *DPB1* that one explanation for this could be that since *HLA* alleles are neither necessary nor sufficient to cause leukaemia they may confer protection to some numerically more important infectious agent. Thus, c-ALL high-risk *HLA* alleles might be less effective in protecting against a leukaemogenic infection caused by molecular mimicry of the susceptibility allele by the infectious agent, but be effective against some other infection. Since the transformation of pre-B progenitor cells into c-ALL requires at least two mutations, it seems unlikely that *HLA* alleles determine whether a fetus will succumb to an *in utero* infection. This might be expected to be associated with birth abnormalities in children with leukaemia.

A different interpretation is suggested by the Greaves hypothesis (Greaves and Alexander, 1993). This postulates that the chance of a second mutation in a preleukaemic pre-B progenitor population is promoted by post-natal infection-induced cell division. *HLA* alleles associated with a risk of c-ALL might in this case be the target of, or induce, the immunostimulatory signals which lead to the increased division of the pre-leukaemic population. If an infectious agent contained immunogenic peptides capable of binding to alleles at more than one *HLA* class II locus, this might maximise immune recognition but also enhance pre-B stimulation. Such a phenomenon might explain the apparent independence of *DPB1*- and *DQB1*-associated susceptibility to c-ALL.

We found a difference in the percentage of boys and girls with *DQB1**0501 ($RR_{M:F}$, 2.92), and *DQB1**0602 ($RR_{M:F}$, 0.24). This sex difference in the frequency of a susceptibility allele was absent in our previous analysis of *DPB1* (Taylor *et al.*, 1995). One explanation for the sex difference could be that there are subtle male–female differences in genetic susceptibility to immune-associated disease as previously suggested (Purtilo, 1979). Since there is an excess of males to female with c-ALL in our series, it is possible that X-linked immunoregulatory genes influence the ability of males and females to respond to *HLA*-associated immunogenic peptides.

Genotype analysis revealed a higher frequency of *DQB1**0201 homozygosity in the controls than in the patients. We can exclude typing artifact due to the misidentification of heterozygotes as homozygotes as both series were typed at the same time, using the same reagents and techniques. We can also exclude increased *HLA* homozygosity due to consanguineous ethnic minority parents, since we excluded Asian infants from the control series. The result suggests that *0201 homozygosity may have a protective effect in c-ALL. It is of interest to note that none of the amino acid sequence motifs found in *0501, or any of the other alleles associated with an increased risk of c-ALL is also found in *0201. More specifically, the serine residue at position 30, and arginine–lysine at positions 70–71 of *0201, both differ from the motifs associated with *DQB1* susceptibility to c-ALL, and may be considered as conferring protection.

The amino-acid motifs associated with an increased risk of c-ALL include glycine$^{26}$, histidine$^{30}$, valine$^{57}$ and glutamic acid$^{66}$–valine$^{67}$. Of the four motifs at position 57, aspartic acid ($Asp^{57}$) is present in only 48% of children with c-ALL compared with 64% of infant controls, and is negatively associated with c-ALL. It has been known for some time that $Asp^{57}$ confers resistance to insulin-dependent diabetes (Todd *et al.*, 1987; Morel *et al.*, 1988; Tosi *et al.*, 1994), although the presence or absence of this motif is not sufficient to account for inherited susceptibility (Thomson *et al.*, 1988; Kockum *et al.*, 1993). Although there are few similarities between c-ALL and IDDM other than their occurrence in childhood, the possibility that a common infectious agent, or similar immunoregulatory defect with a different outcome is involved would repay further study.

**Acknowledgements**
We thank the UKCCCR, the Kay Kendall Leukaemia Fund and the Leukaemia Research Fund for financial support for this work, William Fergusson and Colin Summers for valued assistance, Ruth Carter for secretarial help, and Sandra Roe for graphics.

### References

APPLE RJ, ERLICH HA, KLITZ W, MANOS MM, BECKER TM AND WHEELER CM. (1994). HLA DR-DQ associations with cervical carcinoma show papilloma virus specificity. *Nature Genet.*, **6**, 157–162.

BAISCH JM AND CAPRA JD. (1993). Linkage disequilibrium within the HLA complex does not extend into HLA-DP. *Scand. J. Immunol.*, **37**, 499–503.

BLIN N AND STAFFORD DW. (1976). Isolation of high molecular weight DNA. *Nucleic Acids Res.*, **3**, 2302–2308.

BRESLOW NE AND LANGHOLZ B. (1983). Childhood cancer incidence: geographical and temporal variations. *Int. J. Cancer*, **32**, 703–716.

BUGAWAN TL AND ERLICH HA. (1991). Rapid typing of HLA-DQB1 DNA polymorphism using nonradioactive oligonucleotide probes and amplified DNA. *Immunogenetics*, **33**, 163–170.

CARRINGTON M, MILLER T, WHITE M, GERRARD B, STEWART C, DEAN M AND MANN D. (1992). Typing of HLA-DQA1 and -DQB1 using DNA single strand conformation polymorphism. *Hum. Immunol.*, **33**, 208–212.

COURT BROWN WM AND DOLL R. (1961). Leukaemia in childhood and young adult life. Trends in mortality in relation to aetiology. *Br. Med. J.*, **i**, 981–988.

ERLICH HA AND ARNHEIM N. (1992). Genetic analysis using the polymerase chain reaction. *Annu. Rev. Genet.*, **26**, 479–506.

GREAVES MF AND ALEXANDER FE. (1993). An infectious etiology for common acute lymphoblastic leukemia in childhood? *Leukemia*, **7**, 349–360.

GREAVES MF, PEGRAM SM AND CHAN LC. (1985). Collaborative group study of the epidemiology of acute lymphoblastic leukemia subtypes: background and first report. *Leuk. Res.*, **9**, 715–733.

GREAVES MF, CHAN LC, FORD AM, PEGRAM SM AND WIEDEMANN LM. (1991). Etiological mechanisms in childhood acute lymphoblastic leukemia. In *Childhood Leukemia: Present Problems and Future Prospects,* Proceedings of the Second International Symposium on Children's Cancer, Tokyo, 1989. pp. 3–22. Kluwer: Dordrecht.

KINLEN LJ, CLARKE K AND HUDSON C. (1990). Evidence from population mixing in British new towns 1946–85 of an infective basis for childhood leukaemia. *Lancet*, **336**, 577–582.

KNEALE GW. (1971). Excess sensitivity of pre-leukaemics to pneumonia. A model situation for studying the interaction of infectious disease with cancer. *Br. J. Prev. Soc. Med.*, **25**, 152–159.

KOCKUM I, WASSMUTH R, HOLMBERG E, MICHELSEN B AND LERNMARK A. (1993). HLA-DQ primarilty confers protection and HLA-DR susceptibility in type I (insulin-dependent) diabetes studied in population-based affected families and controls. *Am. J. Hum. Genet.*, **53**, 150–167.

LEE JE, REVEILLE JD, ROSS MI AND PLATSOUCAS CD. (1994). HLA-DQB1*0301 association with increased cutaneous melanoma risk. *Int. J. Cancer*, **59**, 510–513.

LINET MS AND DEVESA SS. (1991). Descriptive epidemiology of childhood leukaemia. *Br. J. Cancer*, **63**, 424–429.

LO YMD, PATEL P, MEHAL WZ, FLEMING KA, BELL JI AND WAINSCOAT JS. (1992). Analysis of complex genetic systems by ARMS-SSCP: application to HLA genotyping. *Nucleic Acids Res.*, **20**, 1005–1009.

MOLKENTIN J, GORSKI J AND BAXTER-LOWE LA. (1991). Detection of 14 HLA-DQB1 alleles by oligotyping. *Hum. Immunol.*, **31**, 114–122.

MOREL PA, DORMAN JS, TODD JA, McDEVITT HO AND TRUCCO M. (1988). Aspartic acid at position 57 of the HLA-DQ $\beta$ chain protects against type I diabetes; a family study. *Proc. Natl Acad. Sci.*, **85**, 8111–8115.

OLERUP O, ALDENER A AND FOGDELL A. (1993). *HLA-DQB1* and -DQA1 typing by PCR amplification with sequence specific primers (PCR-SSP) in 2 hours. *Tissue Antigens*, **41**, 119–134.

ORITA M, IWAHANA H, KANAZAWA H, HAYASHI K AND SEKIYA T. (1989). Detection of polymorphisms of human DNA by gel electrophoresis as a single strand conformation polymorphism. *Proc. Natl Acad. Sci. USA*, **86**, 2766–2770.

PARKIN DM, STILLER CA, DRAPER GJ AND BIEBER CA. (1988). The international incidence of childhood cancer. *Int. J. Cancer*, **42**, 511–520.

PURTILO DT. (1979). X-associated immunocompetence. *Lancet*, **i**, 327.

SALAZAR M, DEULOFEUT R, YUNIS JJ, BING DH AND YUNIS EJ. (1993). A fast PCR-SSP method for HLA-DQ generic typing. *Tissue Antigens*, **41**, 102–106.

STEWART A. (1961). Aetiology of childhood malignancies. Congenitally determined leukaemias. *Br. Med. J.*, **i**, 452–460.

STEWART A AND KNEALE GW. (1969). Role of local infections in the recognition of haemopoietic neoplasms. *Nature*, **223**, 741–742.

SUMMERS CW, FERGUSSON WD, GOKHALE DA AND TAYLOR M. (1992). Donor-recipient bone marrow matching by single stranded conformation polymorphism analysis. *Lancet*, **339**, 621.

TAYLOR GM. (1994). Immunogenetics and the aetiology of childhood leukaemia. *Arch. Dis. Child*, **70**, 77–81.

TAYLOR GM, ROBINSON MD, BINCHY A, BIRCH JM, STEVENS RF, JONES PM, CARR T, DEARDEN S AND GOKHALE DA. (1995). Preliminary evidence of an association between HLA-DPB1*0201 and childhood common acute lymphoblastic leukemia supports an infectious aetiology. *Leukemia*, **9**, 440–443.

THOMSON G, ROBINSON WP, KUHNER MK, JOE S, MacDONALD MJ, GOTTSCHALL JL, BARBOSA J, RICH SS, BERTRAMS J, BAUR MP, PARTANEN J, TAIT BD, SCHOBER E, MAYR WR, LUDVIGSSON J, LINDBLOM B, FARID NR, THOMPSON C AND DESCHAPS I. (1988). Genetic heterogeneity, modes of inheritance, and risk estimates for a joint study of Caucasians with insulin-dependent diabetes mellitus. *Am. J. Hum. Genet.*, **43**, 799–816.

TODD JA, BELL JI AND McDEVITT HO. (1987). HLA-DQ$_\beta$ gene contributes to susceptibility and resistance to insulin-dependent diabetes mellitus. *Nature*, **329**, 599–604.

TOSI G, BRUNELLI S, MANTERO G, MAGALINI AR, SOFFIATI M, PINELLI L, TRIDENTE G AND ACCOLLA RS. (1994). The complex interplay of the DQB1 and DQA1 loci in the generation of the susceptible and protective phenotype for insulin-dependent diabetes mellitus. *Mol. Immunol.*, **31**, 429–437.

TROWSDALE J AND CAMPBELL RD. (1992). Complexity of the major histocompatibility complex. *Eur. J. Immunogenet.*, **19**, 45–55.

UNO H, KAWANO K, MATSUOKA H AND TSUDA K. (1988). HLA and adult T cell leukaemia: HLA-linked genes controlling susceptibility to human T cell leukaemia virus type I. *Clin. Exp. Immunol.*, **71**, 211–216.